# Comparison of Nasal Colonization of Methicillin-Resistant *Staphylococcus aureus* in HIV-Infected and Non-HIV Patients Attending the National Public Health Laboratory of Central Nepal

**DOI:** 10.1155/2018/4508757

**Published:** 2018-12-04

**Authors:** Kalash Neupane, Binod Rayamajhee, Jyoti Acharya, Nisha Rijal, Dipendra Shrestha, Binod G C, Mahesh Raj Pant, Pradeep Kumar Shah

**Affiliations:** ^1^Department of Microbiology, Trichandra Multiple Campus, Tribhuvan University, Kathmandu, Nepal; ^2^National College, Tribhuvan University, Khusibu, Kathmandu, Nepal; ^3^Department of Infectious Diseases and Immunology, Kathmandu Research Institute for Biological Sciences, Lalitpur, Nepal; ^4^Department of Bacteriology, National Public Health Laboratory, Teku, Kathmandu, Nepal; ^5^Department of Microbiology, Kathmandu College of Science and Technology, Kamalpokhari, Kathmandu, Nepal

## Abstract

**Background:**

*Staphylococcus aureus* is a cardinal source of community- and hospital-acquired infection. HIV infection is a well-recognized risk factor for methicillin-resistant *S. aureus* (MRSA) carriage and infection. Intrinsically developed antibiotic resistance has sharply increased the burden of MRSA which is often associated with morbidity and mortality of the patients. Moreover, nasal carriage of *S. aureus* plays a significant role in spread of community-associated (CA) *S. aureus* infections.

**Methods:**

This study was conducted from June 2016 to December 2016 at National Public Health Laboratory (NPHL), Kathmandu, with an aim to assess the rate of *S. aureus* nasal carriage and MRSA carriage among HIV-infected and non-HIV patients. A total of 600 nonrepeated nasal swabs were analyzed following standard microbiological procedures, where 300 swabs were from HIV-infected patients while remaining 300 were from non-HIV patients. The isolates were identified on the basis of colony characteristics and a series of biochemical tests. The antibiotic susceptibility test (AST) was performed by the modified Kirby–Bauer disc diffusion method. Inducible clindamycin resistance in isolates was confirmed by the D-test method.

**Results:**

Overall, out of 600 nasal swabs of patients tested, 125 (20.8%) were *S. aureus* nasal carriers which included 80 out of 300 (26.66%) among HIV-infected patients and 45 (15%) out of 300 among non-HIV patients, and the result was statistically significant (*p*=0.0043). Among the isolated *S. aureus*, 11 (13.8%) MRSA were confirmed in HIV-infected while 3 (6.7%) MRSA were detected from non-HIV patients. A higher number of *S. aureus* carriers was detected among HIV-infected males 40 (26.49%), whereas MRSA carriage was more prevalent among HIV-infected females 7 (5.1%). Among the HIV-infected, patients of age group 31–40 years were the ones with highest carriage rate 36 (45%), while in non-HIV patients, the highest rate 13 (28.9%) of carriage was detected among the patients of age group 21–30 years. Statistically significant difference was found between *S. aureus* carriage and HIV infection in patients (*p* < 0.05). Higher rate 2/3 (66.7%) of inducible clindamycin resistance in MRSA was detected from non-HIV patients in comparison to HIV-infected patients 7/11 (63.63%) while the result was statistically insignificant (*p* > 0.05). All the MRSA isolates (100%) were resistant against co-trimoxazole while ciprofloxacin showed high rate of sensitivity towards both MSSA and MRSA. None of the isolates were detected as VRSA. The major factors associated with nasal colonization of *S. aureus* were close personal contact, current smoking habit, and working or living in a farm (*p* < 0.05).

**Conclusion:**

Regular surveillance and monitoring of MRSA nasal carriage and antibiotic susceptibility pattern are of prime importance in controlling *S. aureus* infections especially in high risk groups like HIV-infected patients.

## 1. Introduction

*Staphylococcus aureus* is the most prevalent pathogen in community and health care setting and has been a serious threat to human health since its discovery [1]. *S. aureus*, especially methicillin-resistant *S. aureus* (MRSA), is responsible for varieties of maladies ranging from folliculitis to food poisoning. Moreover, it is responsible for causing life-threatening infections such as endocarditis, necrotizing pneumonitis, and osteomyelitis, among others [2]. Community-acquired MRSA (CA-MRSA) strain has emerged as a challenging pathogen which is frequently isolated from military personnel, drug users, athletes, and men who have sex with men [3]. The enduring threat and changing nature of *S. aureus* as a leading infectious pathogen has been well reported [4]. It is a common human skin colonizer, and pathogen armed with various classes of virulence factors including pore-forming toxins, superantigens, phagocytosis inhibitors, and biofilm forming capacity [5].

Some of the most severe infections caused by *S. aureus* include bacteremia, pneumonia, osteomyelitis, toxic shock syndrome, acute endocarditis, myocarditis, meningitis and abscesses in muscles, genitourinary tract infection, infection of central nervous system and various intraabdominal organs [6]. Methicillin-resistant *S. aureus* (MRSA) is a predominant cause of infection both in clinical and community settings which has amplified the disease burden to a major public health problem [7]. Colonizing feature of *S. aureus* is a potential factor for infection, an individual colonized with MRSA strain has a two to twelve fold more risk of subsequent infection [8]. Colonization and infection of MRSA has been recognized more in HIV-infected persons [9]. Moreover, higher prevalence of pathogenic MRSA strains have been documented among HIV-infected pediatric patients [10]. HIV infection and young age are considered as independent risk factors of MRSA infection [1]. High nasal carriage rate of MRSA in HIV infected persons may require early interventions. Hospitalized HIV-infected patients are nearly 17 times more likely to get *S. aureus* infection in comparison to non-HIV patients [11]. Not only this, the increasing prevalence of infections caused by MRSA has been difficult to treat due to the high rate of resistance to commonly prescribed antibiotics [12]. Determination of *S. aureus* nasal carriage rate and antibiotic resistance profiles along with molecular typing of nasal *S. aureus* isolates in healthy populations is necessary to identify risk factors associated with *S. aureus* infection [13–15].

In Nepal, reports on nasal carriage rate in vulnerable groups like HIV sero-positive individuals are very limited. Therefore, this study was carried out to determine the rate of nasal carriage of *S. aureus* in HIV-infected patients which was compared with healthy carriers. Furthermore, the antibiotic resistance patterns of isolates to commonly prescribed antibiotics, was also investigated. This study could be beneficial in management of MRSA infections, particularly in HIV-infected patients, while suggesting appropriate measures for controlling the possible risks of *S. aureus* infections in HIV-infected patients.

## 2. Methods and Materials

### 2.1. Study Design

This study was a prospective cross-sectional study. The study was carried out at bacteriology laboratory of National Public Health Laboratory, Teku, Kathmandu. Study population consisted of HIV-infected and non-HIV infected patients from whom nasal swab was collected. A total of 600 nasal swabs were collected for laboratory diagnosis, of which 300 swabs each were from HIV and non-HIV patients. The study duration was 6 months from June 2016 to December 2016. Preformed questionnaire was used to obtain the clinical history and demographic information of each patient. We also collected the information about health conditions and environmental factors of the subjects which were hypothesized to be major factors associated with MRSA colonization in the study population.

### 2.2. Inclusion and Exclusion Criteria

Patients with known history of HIV infection (seropositive) and noninfection (seronegative) were included in the study. Nasal swabs collected with standard operating procedure were accepted where complete label and strict sterile condition was maintained. Those samples that were improperly labeled and those from the patients who were non‐HIV but having other immunodeficiency conditions like renal transplant, cancer, diabetes, liver cirrhosis, malignancy, chronic cardiovascular diseases, or consuming any immunosuppressant medicine were excluded from the study. Additionally, patients who had received any kind of antibiotics within the previous two weeks were also excluded from this study.

### 2.3. Specimen Collection and Transport

Sampling procedure was done by a well-trained laboratory technician. Nasal swabs were collected by using sterile cotton swabs moistened with sterile normal saline. Each nasal swab was obtained by rotating 2–3 times in the anterior nares of patients' and was transported to bacteriology department quickly for further processing [16]. All the collected swabs were transported in a zip lock bag to the laboratory. No transport medium was used because the bacteriology department and sample collection sites were adjacent to each other and additionally, drying of swab was prevented.

### 2.4. Laboratory Analysis

#### 2.4.1. Microscopic Observation

The nasal swabs were evenly smeared on clean, dry, and grease-free glass slide. Then smear was heat fixed and stained by Gram stain procedure [17]. The stained smear was observed microscopically using the 100x objective for presence of Gram positive cocci in grape-like clusters.

#### 2.4.2. Culture of Nasal Swabs

Primary culture was done on 5% sheep blood agar, Mac-Conkey agar, and Mannitol salt agar under aseptic conditions. Identification of isolated colonies was done by colony morphology, Gram staining, catalase test, slide coagulase test, and tube coagulase test [18].

#### 2.4.3. Antibiotic Susceptibility Testing

Antibiotic susceptibility test was performed on Mueller Hinton Agar (MHA) by modified Kirby-Bauer disk diffusion method as per the Clinical Laboratory and Standards Institute 2016 guidelines [19] and the obtained results were interpreted accordingly. The antibiotics discs and concentrations used were ciprofloxacin (5 *µ*g), penicillin G (30 *µ*g), gentamicin (30 *µ*g) co-trimoxazole (25 *µ*g), cefoxitin (30 *µ*g), erythromycin (30 *µ*g), tetracycline (30 *µ*g), clindamycin (2 *µ*g), and vancomycin (30 *µ*g). The diameter of zone of inhibition (ZOI) was measured and the results were interpreted. Antimicrobials doses were selected on the basis of prescription frequency by physician. Minimum inhibitory concentration (MIC) values of used antibiotics were unable to determine due to unavailability of all antibiotics powder at the time of study period.

#### 2.4.4. Screening of Methicillin-Resistant *S. aureus* (MRSA)

Screening for methicillin resistance in *S. aureus* was done by using cefoxitin (30 *µ*g) antibiotic disc following modified Kirby-Bauer disc diffusion technique. Those *S. aureus* that showed zone size of ≤21 mm around cefoxitin disk were confirmed as MRSA strain.

#### 2.4.5. Screening of Inducible Clindamycin-Resistant *S. aureus*

The macrolide-inducible clindamycin resistance in *S. aureus* was detected by D test method [20]. 0.5 McFarland standard bacterial suspensions of the test isolate was inoculated on MHA plate and then clindamycin (2 *µ*g) and erythromycin (15 *μ*g) discs were kept 15 mm edge to edge on the same plate. Then the plates were aerobically incubated overnight at 37°C. After the incubation period, flattening of zone (D-shaped) around the clindamycin disc was considered as inducible clindamycin resistance positive in *S. aureus*. Results of erythromycin-resistant *S. aureus* after D-test were interpreted into three phenotypic categories; MS_B_ (macrolide-streptogramin B), inducible MLS_B_ (_i_MLS_B_), and constitutive _c_MLS_B_ phenotype.

### 2.5. Quality Control

Media preparation, inoculation, and culture were performed in strict aseptic conditions. The prepared media were checked for the appearance of pure growth of organisms by using ATCC control strain. Stable strain of *S. aureus* ATCC 25923 was used as the control organism for the laboratory procedures. The thickness of MHA plates was maintained at 4 mm and the pH at 7.2–7.4. Microscope, incubator, centrifuge, refrigerator, water bath, autoclave, and hot air oven were checked regularly to ensure the correct functioning of equipment for the reliability of results. The temperature for all equipment was monitored and recorded.

### 2.6. Data Analysis

All data were analyzed using SPSS version 21.0. Chi square test was calculated where *p* value of <0.05 was considered statistically significant at 95% of confidence level.

## 3. Results

Among 600 nasal swabs investigated for determination of *S. aureus* and MRSA nasal carriage, 125 patients were found to be colonized with *S. aureus* among which 80 specimens were from HIV-infected and 45 from non-HIV individuals. The total prevalence of *S. aureus* colonization in HIV was found to be 26.66% while colonization in non-HIV was 15%. The MRSA colonization among the HIV patients was 13.8% (11/80) among 80 *S. aureus* isolates, whereas the MRSA colonization in non-HIV was 6.7% (3/45) among 45 *S. aureus* isolates.

Out of 300 nasal swabs analyzed from HIV-infected individuals, *S. aureus* isolates were 80, of which 26.5% (40/151) were male carriers whereas 23.2% (32/138) were female carriers and among third gender 72.7% (8/11) were *S. aureus* carriers (Table 1). Similarly, out of 45 *S. aureus* isolates from the 300 nasal swabs collected from non-HIV patients; 17.5% (27/154) were isolated from males whereas 12.3% (18/146) were from females. The study reveals that the *S. aureus* carrier percentage was higher among males in HIV-infected as well as in non-HIV patients (Table 2). The nasal carriage rate was not statistically significant with gender of patients (*p* > 0.05).

In HIV-infected patients, a high rate of *S. aureus* nasal carriage was found among the patients of age group 31–40 years with 36 (45%) followed by 21–30 years with 15 (18.8%), 41–50 years with 13 (17.5%), 11–20 years with 8 (10%) whereas *S. aureus* was not detected from the patients of age group 61 year or above (Table 1). Similarly, in non-HIV patients the high rate of *S. aureus* nasal carriage was found among the patients of age group 31–40 years with 12 (26.7%) followed by 11 (24.4%) in age group 41–50 years, 7 (15.6%) in age group 51–60 years, 5 (11.1%) in patients of age group 61 year or above whereas *S. aureus* was not detected from the patients of age group 10 years or less (Table 2). *S. aureus* carrier rate was statistically insignificant in relation to age groups of patients (*p* > 0.05).

**Figure 1:**
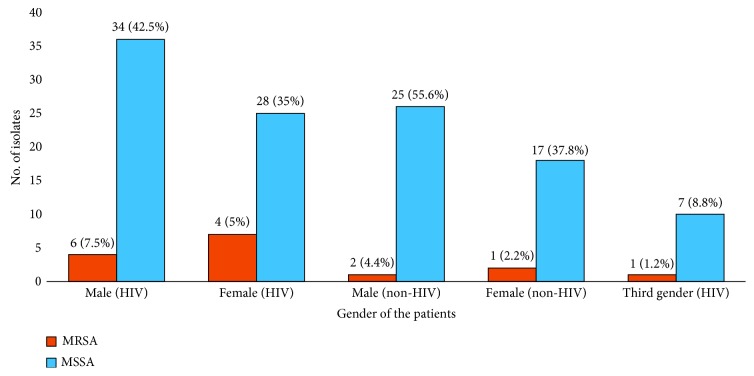
Gender-wise distribution of MRSA and MSSA isolates among HIV-infected and non-HIV patients.

Among the 300 HIV-infected patients, the growth of *S. aureus* was found in 80 (26.6%), of which 11 (13.8%) were confirmed as MRSA strains and remaining 69 (86.2%) were methicillin-sensitive *Staphylococcus aureus* (MSSA) (Table 1). On the other hand, from the 300 non-HIV patients 45 (15%) *S. aureus* were isolated where 3 (6.7%) isolates were confirmed as MRSA strain whereas 42 (93.3%) were MSSA strains (Table 2). The results claim higher percentage of MRSA among HIV-infected patients as compared to non-HIV patients. The association between MRSA isolates and HIV-infected patients was found to be statistically significant (*p* < 0.05). More MRSA isolates were isolated from HIV-infected male patients 6(7.5%) than from females 4 (5%) whereas 1 (1.3%) was isolated from third gender patients with known HIV infection. MSSA was found higher 34 (42.5%) in male patients as compared to female patients 28 (35%). Similarly, more MRSA 2 (4.4%) was found among male non-HIV patients whereas one MRSA was reported from female patients. MSSA isolates rate was also higher among male 25 (55.6%) as compared to female 17 (37.8%) patients (Figure 1). MRSA and MSSA carrier rate was statistically insignificant in relation with gender of patients (*p* > 0.05).

Among the *S. aureus* isolates, the highest rate of antibiotic susceptibility was observed against vancomycin (100%) followed by tetracycline (94.4%), cefoxitin (88.8%) and gentamycin (80.8). High rate of resistance was seen towards erythromycin (41.6%) followed by co-trimoxazole (40.8%). Most 10 (71.4%) of the MRSA were resistant towards ciprofloxacin (Table 3).

Among 125 *S. aureus* isolates from HIV-infected and non-HIV patients, percentage of both inducible and constitutive clindamycin resistance was higher in MRSA as compare to MSSA strains. Inducible MLS_B_-(iMLS_B_) resistance, constitutive MLS_B_ resistance, and macrolide–streptogramin B (MS_B_) resistance were detected in 30 (24%), 19 (15.2%), and 34 (27.2%) isolates of *S. aureus*, respectively. Higher rate of iMLS_B_ and cMLS_B_ was seen among *S. aureus* isolated from HIV-infected patients while the rate of inducible MLS_B_ resistance was statistically insignificant as to the origin of *S. aureus* isolates i.e. HIV-infected and non-HIV patients (*p* > 0.05) (Table 4).

Nasal colonization rate of *S. aureus* was found significantly higher among the patients who used to work or live on a farm (*p* < 0.05). Similarly, inpatients showed higher rate 69 (55.2%) of *S. aureus* carriage as compared to outpatients 56 (44.8%), which was statistically significant data (*p*=0.005). Additionally, patients with current smoking habit showed higher rate 76 (60.8%) of *S. aureus* carriage as compare to non-smokers 49 (39.2%) (*p*=0.0051). Patients who had a job with close human contact were more prone to being a *S. aureus* carrier 89 (71.2%) as compared to those not having close human contact jobs 36 (28.8%) (*p*=0.0076) (Table 5).

## 4. Discussion

Colonization of MRSA significantly increases the risk of *S. aureus* infection during hospital stay and even after discharge [21]. Furthermore, nasal carriage of *S. aureus* is a cardinal source of infection and nasopharynx is considered as the main reservoir of *S. aureus* [1]. The outbreaks of MRSA infection especially in group of people like security shelters, health care centers and refugee camps strongly supports the theory that the spread of *S. aureus* requires close personal contact [22]. The rate of nasal carriage depends on various factors like clinical environment, geographical area, occupation of patients, and patient's immune status among others. Previous studies have reported up to 33% nasal colonization of *S. aureus* among HIV-infected population [12].

Total of 600 nasal swabs were analyzed in this study where 300 nasal swabs were from HIV-infected patients and remaining 300 were from non-HIV patients. The prevalence of *S. aureus* in HIV-infected patients was found to be more than the rate in non-HIV patients. This study shows the total prevalence rate of nasal carriage of *S. aureus* to be 20.8% (125/600). HIV-infected patients have higher rate of *S. aureus* nasal carriage than non-HIV infected patients and the result was statistically significant (*p* < 0.05). High rate of *S. aureus* colonization in HIV infected individuals can lead to severe infections in this group of people [23]. Immune status along with an individual behavioral features can play a positive role in *S. aureus* colonization and subsequent infection [24]. In a similar study carried out by Gonsu et al. (2013) in Cameroon, 40.6% of *S. aureus* nasal carriage was reported, whereas 34.6% of isolated *S. aureus* were MRSA among medical staffs and adult hospitalized patients [25]. Lilian et al. (2013) from Brazil have published a report of 27.2% as the prevalence of nasal colonization with *S. aureus* in patients with HIV/AIDS whereas the rate of MRSA was 21.8% [26]. Furthermore, the study has reported that nasal colonization of *S. aureus* was found to be more among the patients with CD4 cell count <200 cells/mm^3^ and who had a high HIV viral load. Hence, HIV-infected patients are more vulnerable group to develop infections due to MRSA. On the other hand, *S. aureus* nasal colonization rate was found 18.4% among the healthy secondary school going students in Iraq whereas only 2.04% isolates were found to be MRSA [27].

The nasal carriage rate of *S. aureus* can be directly linked with underlying diseases, surrounding environment, age of patients and close contact activities [28]. In a study conducted among health care workers of Pakistan, 18.2% were nasal carriers of *S. aureus* where the count was lead my midwives (30%) then maintenance staffs of the hospital (28.6%) where only 1.5% of isolates were MRSA [29]. The real reason behind higher rate of *S. aureus* nasal colonization in midwives is unclear but this could be due to frequent contact with hospitalized patients and longer stay in hospital environment [30]. In a result reported by Khanal et al. (2015) the highest rate of MRSA nasal carriage was noted among hospital nurses which was 7.8% followed by hospital staffs of surgical wards and operating department whereas high number of *S. aureus* isolates (20.8%) were reported from doctors [31].

This study reveals higher rate of *S. aureus* colonization among patients of age group 31–40 years both in HIV-infected and no-HIV patients with prevalence rate 45% and 26.7%, respectively. Similarly, in a study carried out by Reinato et al., they have reported 36.1% of *S. aureus* colonization rate among the HIV/AIDS patients of age group 30–39 years [26]. The risk factors that have been associated with the emergence of MRSA infections among HIV infected individuals are not practically understood well but may be due to some possible risk factors, such as living conditions, prior hospitalization, use of fluoroquinolones and third generation cephalosporin antibiotics, intravenous drug usage, other secondary coinfections and low level of CD4 cells [32]. Some studies have suggested that participation in high risk sexual behaviors, use of public bath, anal intercourse, men who have sex with men (MSM), and sex with multiple partners may flourish the transmission of *S. aureus* and MRSA [33–35].

In this study, more *S. aureus* isolates were reported from males as compared to females both in HIV-infected and non-HIV patients though the result was not statistically significant (*p* < 0.05). The nasal carriage of *S. aureus* is found to be higher among male of active age groups who are mostly involved in outdoor activities, frequently visit to health care centers, and regular exposure to mass of people [31]. In contrast, higher rate of *S. aureus* nasal colonization was reported in female (64%) than in male (36%) patients from Iran [36]. Patients with close contact job were more MSSA carriers 71.2% (89/125), as compared to 28.8% (36/125) patients who had no close contact and the result was statistically significant (*p*=0.0076). Similar result was reported by Oliva et al. where 57% of people who had close contact job were MSSA carriers and 27.1% subjects were MSSA carriers who did not have human contact job [1]. This study shows higher rate of MSSA carriers among patients with current smoking habit (*p*=0.0051) and patients who used to work or live in farm area (*p* < 0.05). Similarly, there were more MSSA carriers among inpatients (55.2%) in comparison to out-patients (44.8%). The association between type of patients (inpatients/out-patients) and MSSA carriers rate was statistically significant (*p*=0.005). These results suggest that alcohol consumption, working/living in farm area and job with human contact environment positively contribute towards the spread of MSSA and MRSA. Moreover, hospitalized patients are at greater risk of having CA-MRSA colonization. Individual behavioral factors, environmental, social, HIV-host factors, and all these in united form play a positive role in *S. aureus* colonization and subsequent series of infection [30].

In this study, 94.4% (118/125) of *S. aureus* isolates were sensitive to tetracycline followed by cefoxitin 88.8% (111/125), whereas 9.2% (14/125) of isolates were resistant to cefoxitin. High rate of resistance 41.6% was observed against the antibiotic erythromycin followed by co-trimoxazole (40.8%), ciprofloxacin (25.6%) and penicillin G (23.2%), respectively. There was 100% sensitivity to the antibiotic vancomycin. The rate of co-trimoxazole resistance was higher among *S. aureus* isolated from HIV-infected patients than non-HIV patients. Co-trimoxazole is being used as a major chemoprophylaxis agent for HIV-infected patients for the treatment of various bacterial infections. Increasing rate of antibiotic resistance among MRSA isolates may increase the burden of infections in the community and clinical setting especially in patients with HIV/AIDS [37]. Similar result was reported on antibiotic susceptibility pattern of *S. aureus* isolated from health care workers of western Nepal by Khanal et al. (2015) where 93.8% isolates were sensitive to tetracycline and 78.1% were sensitive to cefoxitin whereas 100% MRSA was penicillin resistant [31].

Among 125 *S. aureus* isolates, 24% (30/125) were detected to have inducible clindamycin (iMLS_B_) resistance whereas high rate of iMLS_B_ isolates were confirmed from MSSA 70% (21/30) than MRSA 30% (9/30). Additionally, 15.2% (19/125) constitutive MLS_B_ and 27.2% (34/125) macrolide-streptogramin B (MS_B_) resistance *S. aureus* were detected where 33.6% (42/125) of *S. aureus* isolates were clindamycin sensitive. None of the MRSA isolates were clindamycin sensitive. The results are not consistent with the findings reported by Prabhu et al. (2011) where 37.52% and 16.66% of *S. aureus* isolates from non-HIV patients were confirmed as iMLS_B_ and cMLS_B_ resistance, respectively [38]. The findings of this study show that D-test should be performed in routine antibiotic susceptibility test which could guide the appropriate treatment options during the infection caused by *S. aureus* and MRSA. The rate of iMLS_B_ resistance of this study is higher as compared to previous reports by Ansari et al. and Adhikari et al. where they have reported 12.4% [39] and 10% [40] of inducible clindamycin-resistant *S. aureus*, respectively. This variation could be due to different clinical settings, type of patients, environment, *S. aureus* with different susceptibility patterns, and behavioral factors of patients [41]. Clindamycin is a drug of choice for the treatment of *S. aureus* infections but constitutive or inducible resistance to this antibiotic has been a major cause of treatment failure. Both MRSA and inducible clindamycin resistance *S. aureus* are serious challenge to health care management of Nepal especially in immunosuppressed groups like patients with HIV/AIDS, tuberculosis, renal transplant, cancer, diabetes, liver cirrhosis, malignancy, and chronic cardiovascular diseases.

High prevalence of nasal colonization along with increased rate of antibiotic resistance in *S. aureus* has posed a great threat to public health of the country and beyond. Compromised immune system and other risk factors including behavioral characters of HIV patients have led to emergence of MRSA which ultimately results in increase burden of community and hospital acquired infections. Increasing treatment failure due to high level of antibiotic resistance of *S. aureus* has expanded the burden of diseases in developing countries. Routine surveillance, proper monitoring of antibiotic resistance pattern and implementation of control strategies to prevent circulation of *S. aureus* strains in both clinical and hospital settings along with early detection of pathogenic MRSA isolates is very crucial for the reduction of morbidity and mortality due to diverse forms of *S. aureus* infections. Hence, routine screening, investigation of *S. aureus* nasal carriage particularly in immune deficient individuals, monitoring of their antibiotic susceptibility and control of *S. aureus* transmission are preventive me2asures for proper management of infections due to *S. aureus*.

## 5. Conclusion

The findings of this study show high rates of *S. aureus* colonization (mainly MRSA) and subsequent infections among HIV-infected patients. Smoking and alcohol drinking habits pose an individual at higher risk of *S. aureus* infection. Inducible clindamycin resistance (iMLS_B_) *S. aureus* in HIV patients is increasing. D-test for the detection of iMLS_B_ can be included as a screening test in routine laboratory investigation. Special attention is urgent to keep transmission and emergence of MRSA strains under control in people living with HIV/AIDS. *S. aureus* isolates showed high frequency of sensitive to tetracycline but we should take in account geographic differences in AST pattern when selecting antibiotic. Control strategies and interventions are very crucial to stop the spread to this “superbug” beyond the border.

## 6. Limitations

We cannot reveal the exact figure of *S. aureus* colonization without investigating swabs of other probable body sites like groin, pharynx, axillae, and anus. Furthermore, we could not collect the other epidemiological risk factors which could directly affect the MSSA and MRSA colonization and infection pattern. We could not perform/provide the CD4 count data to correlate with the immune status of HIV infected individuals too. Due to limitation of laboratory resources we could not run molecular procedures for confirmation of mecA gene in MRSA isolates. Further investigation in molecular level with collection of broad risk factors of large number of participants from different parts of the nation is required to generalize the result.

## Figures and Tables

**Table 1:** Socio demographic characteristics of the HIV-infected patients and ratio of *S. aureus* carriage.

Demographic features	Type of patients	*S. aureus* (no. (%))	MRSA (no. (%))	No growth	Total (no. (%))
Gender	HIV-infected				
Male	151	40 (50)	6 (7.5)	111 (50.4)	151 (50.3)
Female	138	32 (40)	4 (5)	106 (48.2)	138 (46)
Third gender	11	8 (10)	1 (1.3)	3 (1.4)	11 (3.7)
*Total*	**300**	**80 (26.7)**	**11 (13.8)**	**220 (73.3)**	**300 (100)**
Age in years					
≤10	18	4 (5)	0	14 (77.8)	18 (6)
11–20	29	8 (10)	2 (2.5)	21 (72.4)	29 (9.7)
21–30	59	15 (18.8)	3 (3.8)	44 (74.6)	59 (19.7)
31–40	93	36 (45)	5 (6.3)	57 (61.3)	93 (31)
41–50	76	13 (17.5)	1 (1.3)	63 (82.9)	76 (25.3)
51–60	21	4 (5)	0	17 (80.9)	21 (7)
≥61	4	0	0	4 (100)	4 (1.3)
*Total*	**300**	**80 (26.7)**	**11 (13.8)**	**220 (73.3)**	**300 (100)**

**Table 2:** Socio demographic characteristics of the non-HIV patients and ratio of *S. aureus* carriage.

Demographic features	Type of patients	*S. aureus* (no. (%))	MRSA (no. (%))	No growth	Total (no. (%))
Gender	Non-HIV patients				
Male	154	27 (60)	2 (4.4)	127 (49.8)	154 (51.3)
Female	146	18 (40)	1 (2.3)	128 (50.2)	146 (48.7)
Third gender	0	0	0	0	0
*Total*	**300**	**45 (15)**	**3 (6.7)**	**255 (85)**	**300 (100)**
Age in years					
≤10	16	0	0	16 (100)	16 (5.3)
11–20	33	4 (8.9)	0	29 (87.9)	33 (11)
21–30	45	6 (13.3)	0	39 (86.7)	45 (15)
31–40	101	12 (26.7)	2 (4.4)	89 (88.1)	101 (33.7)
41–50	66	11 (24.4)	1 (2.2)	55 (83.3)	66 (22)
51–60	26	7 (15.6)	0	19 (73.1)	26 (8.7)
≥61	13	5 (11.1)	0	8 (61.5)	13 (4.3)
*Total*	**300**	**45 (15)**	**3 (6.7)**	**255 (85)**	**300 (100)**

**Table 3:** Antibiotic susceptibility pattern of MSSA and MRSA bacteria isolated from HIV-infected and non-HIV patients.

Isolates	Type of patients	RXN	Antimicrobial agents (no. (%))							
			CX	CIP	COT	GEN	VA	P	E	TE
MSSA (*n*=69)	HIV-infected	S	69 (100)	56 (81.2)	45 (65.2)	60 (86.9)	69 (100)	57 (82.6)	41 (59.4)	66 (95.7)
		R	0	13 (18.8)	24 (34.8)	9 (13.1)	0	12 (17.4)	28 (40.6)	3 (4.3)
MSSA (*n*=42)	Non-HIV	S	42 (100)	33 (78.6)	24 (57.1)	36 (85.7)	42 (100)	39 (92.38	27 (64.3)	42 (100)
		R	0	9 (21.4)	18 (42.9)	6 (14.3)	0	3 (7.2)	15 (35.7)	0
MRSA (*n*=11)	HIV-infected	S	0	3 (27.3)	5 (45.5)	3 (27.3)	11 (100)	0	4 (36.4)	8 (72.7)
		R	11 (100)	8 (72.7)	6 (54.5)	8 (72.7)	0	11 (100)	7 (63.6)	3 (27.3)
MRSA (*n*=3)	Non-HIV	S	0	1 (33.3)	0	2 (66.7)	3 (100)	0	1 (33.3)	2 (66.7)
		R	3 (100)	2 (66.7)	3 (100)	1 (33.3)	0	3 (100)	2 (66.7)	1 (33.3)
*Total (n*=125)		**S**	**111 (88.8)**	**93 (74.4)**	**74 (59.2)**	**101 (80.8)**	**125 (100)**	**96 (76.8)**	**73 (58.4)**	**118 (94.4)**
		**R**	**14 (9.2)**	**32 (25.6)**	**51 (40.8)**	**24 (19.2)**	**0**	**29 (23.2)**	**52 (41.6)**	**7 (5.6)**

Key: R = resistant, S = sensitive, RXN = reaction, CX = cefoxitin, CIP = ciprofloxacin, COT = co-trimoxazole, GEN = gentamicin, VA = vancomycin, P = penicillin G, E = erythromycin, TE = tetracycline.

**Table 4:** Clindamycin resistance among MRSA and MSSA strains.

Susceptibility pattern (phenotypes)	E	CD	*D*-test	MSSA		MRSA		Total *S. aureus*
				HIV-infected (*n*=69) (no. (%))	Non-HIV (*n*=42) (no. (%))	HIV-infected (*n*=11) (no. (%))	Non-HIV (*n*=3) (no. (%))	(*n*=125) (no. (%))
Inducible MLS_B_-(iMLS_B_)	R	S	Positive	17 (24.6)	4 (9.5)	7 (63.6)	2 (66.7)	30 (24)
Constitutive MLS_B_-(cMLS_B_)	R	R	Negative	11 (15.9)	5 (11.9)	3 (27.3)	0	19 (15.2)
MS_B_	R	S	Negative	21 (30.4)	11 (26.2)	1 (9.1)	1 (33.3)	34 (27.2)
Susceptible	S	S	Negative	20 (28.9)	22 (52.4)	0	0	42 (33.6)

Key: R = resistant, S = sensitive, E = erythromycin, CD = clindamycin, MS_B_ = macrolide–streptogramin B, MLS_B_= macrolide–lincosamide–streptogramin B.

**Table 5:** Nasal colonization of *S. aureus* with respect to risk factors.

Environmental parameters	*S. aureus* carriers (*n*=125) (no. (%))	*S. aureus* non-carriers (*n*=475) (no. (%))	*p* value
Farm working/living			
Yes	42 (33.6)	306 (64.4)	**<0.05**
No	83 (66.4)	169 (35.6)	
Job conditions			
With close contact	89 (71.2)	276 (58.1)	**0.0076**
Without close contact	36 (28.8)	199 (41.9)	
Current smoking habit			
Yes	76 (60.8)	222 (46.7)	**0.0051**
No	49 (39.2)	253 (53.3)	
Alcoholic/nonalcoholic (current)			
Alcoholic	69 (55.2)	234 (49.3)	**0.319**
Nonalcoholic	56 (44.5)	241 (50.7)	
Outpatient/inpatient			
Outpatient	56 (44.8)	279 (58.7)	**0.005**
Inpatient	69 (55.2)	196 (41.3)	

## Data Availability

The data used to support the findings of this study are included within the article.

## Abbreviations

BA: Blood agar
CA: Chocolate agar
CDC: Centers for Disease Control and Prevention
MA: Mac-Conkey agar
MHA: Mueller Hinton agar
MDR: Multidrug resistance
MRSA: Methicillin-resistant *S. aureus*
CLSI: Clinical Laboratory Standards Institute
HIV: Human immunodeficiency virus
AIDS: Acquired immunodeficiency syndrome
ART: Antiretroviral therapy
AST: Antibiotic susceptibility testing
SCCmec: Staphylococcal cassettes chromosome
SOP: Standard operating procedure
PVL: Panton-Valentine Leukocidin
SSTI: Skin-and-soft tissue infection
NPHL: National Public Health Laboratory
WHO: World Health Organization
VRSA: Vancomycin-resistant *S. aureus*.
